# Molecular translocation between parasitic plants and their hosts

**DOI:** 10.3389/fpls.2025.1716304

**Published:** 2025-12-08

**Authors:** Yuchao Chen, Huilan Wu, Jie Cai, Shenghu Guo, Xiaoyan Gan, Xuan Liu, Jianguo Yang

**Affiliations:** 1 Agricultural Biotechnology Center, Ningxia Academy of Agriculture and Forestry Sciences, Yinchuan, China; 2 National Resource Center for Chinese Meteria Medica, Chinese Academy of Chinese Medical Sciences, Beijing, China

**Keywords:** parasitic plants, horizontal gene transfer, molecular translocation, haustorium, *Cuscuta*

## Abstract

Parasitic plants are a special group deriving their nutrients from another plant, some of which such as witchweeds (*Striga* spp.) and broomrapes (*Orobanche* and *Phelipanche* spp.) are referred as weeds responsible for severe crop losses in agriculture. The parasite attaches to and feeds off its host using a haustorium, which also facilitates the transport of various molecules between the parasite and its host. These translocation molecules have received extensive attention from researchers. In this review, we summarize the existing knowledge on the transfer of molecules such as pathogens, herbicides, RNAs, and proteins between parasitic plants and their hosts, and discuss their potential implications. Additionally, we provide an overview of horizontal gene transfer (HGT) between species, which is particularly evident in the mitochondrial and nuclear genomes, with some transgenes assumed to have functional roles in their recipient species, offering new insights into the evolution of parasitic plants. Finally, we discuss the significance of parasitic plant research and the development of future research technologies to advance our understanding of plant parasitism.

## 1 Introduction

Plant parasitism is a complex ecological phenomenon in which parasitic plants absorb various nutrients, such as mineral elements, amino acids, sugars, and intermediate metabolic products, from the host (typically animals or other plants) through their haustorium (Clarke et al., 2019). The haustorium can penetrate the stem or root of the host and establish a connection with the host’s vascular system (Aguilar-Venegas et al., 2023; Yoshida et al., 2016; Ibarra-Laclette et al., 2022). These molecular translocation not only involve the transfer of nutrients but also the transmission of microorganisms such as viruses, viroids, and phytoplasmas. This process forms the core bidirectional regulatory mechanism in the plant parasitism system.

In recent years, with the advancement of molecular biology techniques, significant progress has been made in the study of molecular communication mechanisms between parasitic plants and their hosts. Studies have found that parasitic plants release specific chemicals or signaling molecules to interact with the host cell surface or receptor proteins, thereby regulating the host’s gene expression and physiological activities (Soyemi et al., 2018; Jhu and Sinha, 2022). Nevertheless, there are still many unresolved mysteries regarding the molecular communication mechanisms between parasitic plants and their hosts. For example, how the haustorium specifically recognizes and selects the nutrients it needs to absorb, and the specific role of macromolecule transfer in the parasitism process, remains unclear (Kirschner et al., 2023).

Approximately one percent of angiosperms are parasitic plants thriving by infecting other plants (Nickrent et al., 1998). These parasitic plants have attracted significant research attention due to their adverse impact on crops (Albanova et al., 2023). For instance, many members of parasitic plants, in particular *Striga* spp. (witchweeds), *Orobanche* spp. (broomrapes), and *Cuscuta* (dodder) that parasitize some important food, forage grains, and legumes, are referred as parasitic weeds, which pose a substantial threat to global crop productivity (Parker, 2012). It is estimated that *Striga* and *Orobanche* species infest upwards of 60 million hectares of farmland worldwide, resulting in billions of dollars of losses each year (Parker, 2012; Chesterfield et al., 2020). Eliminating threats of parasitic weeds requires knowledge of the machinery underlying the molecular translocation. Understanding these molecular translocation mechanisms not only helps to elucidate the complex relationship between plants and hosts but also provides a theoretical foundation for developing new biocontrol strategies. Here, we provide a comprehensive review of the current understanding of molecular translocation between the parasite and the host, which is of great significance for controlling parasitic weeds and revealing the coevolution between the parasite and the host.

## 2 Haustorium

One of the key characteristics of parasitic plants is the haustorium, which is involved in the interaction between the host and the parasite. A haustorium is the unique organ that invades host tissues and establishes vascular connections. The haustorium is a specialized organ unique to parasitic plants, playing a crucial role in facilitating parasitism. It penetrates the host’s tissues to establish vascular connections, allowing for the direct acquisition of water, inorganic salts, and organic nutrients (Yoshida et al., 2016). Additionally, the haustorium functions as a bidirectional transport system, mediating the movement of biomolecules such as mRNA, proteins, secondary metabolites, and even pathogens between the parasitic plant and its host. These multifaceted functions enable the parasitic plant to establish a successful parasitic relationship with its host, ensuring its survival and growth. The **Table 1** shows the diameters of the haustoria for different parasitic types.

**Table 1:** Different haustoria of parasitic plants.

Parasitic plant groups	Parasitic type	Diameter range of haustoria	Biomolecular transport	References
*Cuscuta* spp.	Holoparasitic plants	∼100 µm	Transferring phloem-mobile compounds—such as sucrose, amino acids, and phytohormones—from the host plant to the parasitic plant.	(Birschwilks et al., 2006; Shen et al., 2023)
*Orobanche* spp.	Root holoparasite	∼500 µm	Efficient transport of water/ions	(Joel, 2013; Cui et al., 2016)
*Striga* spp.	Root hemiparasite	∼500 µm	Hormone (auxin) directed transport	(Furuta et al., 2021)

## 3 Pathogen translocation in host-parasite

### 3.1 Viruses (Viroids) translocation

Viruses (Viroids) translocation has been documented between host and parasite (Jhu and Sinha, 2022). For example, Hosford (1967) verified that 56 viruses could be translocated from an infected to a healthy host plant via *Cuscuta* (dodder) bridges. A more in-depth research shown that potato Y virus could be transferred between two cultivated tobacco plants through an open phloem connection between *Cuscuta* and its hosts, while only a small amount of virus accumulation was retained in parasite, indicating that the virus did not propagate in parasite during the transfer process (Birschwilks et al., 2006). Therefore, *Cuscuta* probably works for a passive pipeline for viruses translocation between different host plants. Besides *Cuscuta* sp., Gal-on et al. (2009) found *Cucumber mosaic virus* (CMV), *Tomato mosaic virus* (ToMV), *Potato virus Y* (PVY), and *Tomato yellow leaf curl virus* (TYLCV) could translocate from infected host plants to the parasit *Phelipanche aegyptiaca*, and confirmed CMV could replicate in parasite as well as in host, while ToMV, PVY, TYLCV could not replicate in parasite. In addtion, *Potato* sp*indle tuber viroid* (PSTVd) could translocate from the host tomato to parasite *Orobanche ramosa* and replicate in parasite (Vachev et al., 2010). **Table 2** presents some of the viruses that have been discovered in parasitic plants.

**Table 2:** Examples of plant viruses, detected in parasitic plants.

Species	Parasitic plant	Main host	Reference
Little cherry virus	*Cuscuta europea*	Tobacco	(Jelkmann et al., 2010)
Mesta leaf curl virus	*Cuscuta* spp.	Mesta	(Ambuja et al., 2018)
Potato stem mottle virus	*Cuscuta* sp.	Tobacco	(Singh et al., 2020)
Beet curly top virus	*Cuscuta* sp.	Sugar beets	(Singh et al., 2020)
Potato virus Y	*Cuscuta reflexa*	Tobacco	(Birschwilks et al., 2006)

It was discovered that the weeds (such as *Solanum viarum*) in the Nigerian chili fields could simultaneously carry CMV, PVY and ToMV. 83.3% of the weed samples were found to carry at least one of these viruses. Among them, the CMV carrying rate of *Ageratum* conyzoides was as high as 67%, confirming that it serves as a reservoir of viruses for secondary transmission in the field (Arogundade et al., 2021). Recently, a new host adaptation phenomenon has been discovered. Kumar et al (Kumar et al., 2024). found that the Croton yellow *vein mosaic virus* (CYVMV), which originally infected weeds (in the Euphorbiaceae family), was first detected in turnips (*Brassica rapa*). Phylogenetic analysis showed that the virus recombined from the *Synedrella* virus strain (with 85% sequence homology), proving that the parasitic plants facilitated the virus’s cross-species adaptive evolution.

### 3.2 Phytoplasma translocation

Phytoplasmas are cell wall-less plant pathogenic bacteria colonizing in the phloem, which can be translocated by phloem-feeding insects or by vegetative propagation. Parasitic plants can acquire phytoplasmas from their hosts and are served as the vectors for propagation of phytoplasmas. A phytoplasma named alder yellows (ALY), a phytoplasmal aetiology infecting *Alnus* sp*ecies*, was confirmed to have potential to translocate to healthy secondary host *Catharanthus roseus* (periwinkle) via parasite dodder (*Cuscuta odorata*) from naturally infected alder trees (Marcone et al., 1997). Similarly, translocation of phytoplasmas American ALY, pear decline, European stone fruit yellows as well as rubus stunt occurred from infected plants to the experimental host periwinkle by dodder (Kamińska and Korbin, 1999). Moreover, the rate of phytoplasmas translocation probably depended on both phytoplasma type and *Cuscuta* vector type: for example, rubus stunt and cotton phyllody were transmitted at high efficiency, whereas the other phytoplasmas were transmitted at a low rate by *Cuscuta europea* and *Cuscuta campestris*, respectively (Marcone et al., 1999).

The phytoplasmas can secrete various effector proteins to manipulate the host plant’s development processes, hormone signaling, immune system, and so on, in order to achieve their parasitism, transmission, and survival (Oshima et al., 2023), several effectors and function are shown in **Table 3**.

**Table 3:** The function of secreting effectors of phytoplasmas.

Effector	Function	Reference
TENGU	Suppresses auxin signaling, increases production of stems and induces sterility	(Minato et al., 2014)
SAP05	Degrades SPL/GATA via ubiquitin-independent pathway and increases production of stems	(Huang et al., 2021)
SWP16	Inhibits RNA silencing in plant	(Wang et al., 2021)
SWP12	Degrades TaWRKY74 via ubiquitin-dependent pathway and weakens plant resistance	(Bai et al., 2022)

## 4 Herbicides translocation in host-parasite

Herbicides are used as a chemical control of parasitic weed, and there is no significant inhibition effect since the greater part of the parasitic weed life cycle occurs underground (Aly, 2012; Zagorchev et al., 2021). Thus, to effectively control a parasitic weed, herbicide must be taken up by the host that habored natural or genetically induced resistance to herbicide and translocated through the host to the weed. Aviv et al. (2002) reported that transgenic acetolactate synthase-resistant host carrot (*Daucus carota*) treated by herbicide imazapyr allowed for translocation of undegraded imazapyr from host to the parasite *P. aegyptiaca*, leading to the parasite growth suppression. Díaz-Sánchez et al. (2002)demonstrated that translocation of the radioactive herbicide, such as [^14^C]pronamide, [^14^C]glyphosate, and [^14^C]imazapyr from host sunflower (*Helianthus annuus* L.), to parasite *Orobanche cumana* occurred in *Orobanche cumana*-sunflower system, and found that the translocation and deposit of herbicide in parasite was affected by herbicide type, herbicide treatment method, and host growth stage being treated.

Besides, another herbicide chlorsulfuron could kill the parasite *Orobanche ramosa* at tubercular phase by translocating from host, a transgenic tobacco (*Nicotiana tabacum*) harboring *ahas3R* gene expressed high resistance to chlorsulfuron (Slavov et al., 2005). Furthermore, Shilo et al. (2016) revealed that the mechanism of action of the herbicide glyphosate translocated from host tomato (*Solanum lycopersicum*) into parasite *P. aegyptiaca*, and found that despite its total reliance on its host plant, *P. aegyptiaca* suffered from a deficiency of aromatic amino acids caused by the herbicide, which was the same as in host. Given the inhibitory effect of herbicides on the growth of parasitic plants, the way of herbicides translocation between host and parasitic plants provide a new perspective for the control of parasitic weeds.

## 5 DNA translocation in host-parasite

DNA translocation in host-parasite involving three DNA-containing cellular compartments, such as mtDNA (mitochondrial DNA), nuDNA (nuclear genome) and cpDNA (chloroplast DNA), is referred as one of route for HGT (horizontal gene transfer—the exchange of genetic materials between distantly related, non-mating organisms), which is inferred from multigene phylogenetic analysis (Davis and Wurdack, 2004; Yang et al., 2019).

In comparison with both nuDNA and cpDNA, mtDNA allow for higher frequency of translocation due to their ability of being uptaken actively (Koulintchenko et al., 2003; Davis and Xi, 2015) *Rafflesiaceae* (*Rafflesia arnoldii*), an endophytic holoparasite classified in Malpighiales, produces the largest flowers in the world, however, it is incapable of photosynthesis and thus depend exclusively on its host, *Tetrastigma* (Vitaceae), for nutrition. The multigene phylogenetic analyses based on mitochondrial (*matR*) and nuclear loci (18S ribosomal DNA and *PHYC*) showed that Malpighiales was assigned to *Rafflesiaceae*, however, based on the mitochondrial *nad1B-C*, they were grouped within Vitaceae, near their obligate host, *Tetrastigma*. These discordant phylogenetic hypotheses strongly suggested that mtDNA translocation occurred in *Rafflesiaceae* from their hosts (Davis and Wurdack, 2004). Moreover, research revealed that up to 40% of the mtDNA in the parasitic plant species Rafflesiaceae was acquired from its hosts via the mtDNA translocation (Xi et al., 2013). More recently, mtDNA translocation in another parasitic plants *Lophophytum mirabile* parasitizing *Santalales* were also authenticated (Sanchez-Puerta et al., 2017).

In addition to mtDNA, cpDNA translocation has also been demonstrated between the holoparasitic plant *Cistanche deserticola* and its host *Haloxylon ammodendron*. The cpDNA of *C. deserticola* exhibits significant reduction, having lost most genes associated with photosynthesis. However it appears to have regained some functionality by acquiring two copies of the gene *rpoC2* (DNA-dependent RNA polymerase) from *H. ammodendron* (Li et al., 2013).

Although an increasing number of studies on mtDNA or cpDNA translocation in host-parasite interactions are emerging, the translocation of nuDNA remains largely unexplored. *Striga hermonthica*, belonging to the eudicot *Orobanchaceae* family in the order Lamiales, specifically parasitizes monocot plants such as sorghum (*Sorghum bicolor*) and rice (*Oryza sativa*). One gene, designated as *ShContig9483* in *S. hermonthica*, was found to share high similarity with genes in sorghum and rice, but it lacks homologs present in eudicots. This was determined by identifying monocot-specific genes in the S. hermonthica genome through large-scale expressed sequence tag analysis. These findings suggest that *ShContig9483* likely originated from monocots such as sorghum, rice, or other related species, and was subsequently translocated into S. hermonthica (Davis and Xi, 2015). A representative case of nuDNA translocation is the acquisition of the *strictosidine synthase-like (SSL)* gene by parasites from Brassicaceae (Zhang et al., 2014). Both the root parasite *Orobanche aegyptiaca* and the stem parasite *Cuscuta australis* harbour *SSL* copies that exhibiting markedly higher sequence identity with Brassicaceae homologs than with any sequences from their own eudicot relatives.

Interestingly, these translocated DNA in parasite probably execute certain functions (**Table 4**). For example, Zhang et al. (2014) reported the expression levels of the translocated *SSL* genes in *O. aegyptiaca* and *C. australis* varied in different developmental stages and organs, moreover, the *SSL* gene in *C. australis* was inducible after wounding. Yang et al. (2016) suggested that the functions of translocated DNA in parasites were likely related to the development of haustorium, defense against infections, insect toxins, or transcription-related enzymes. Additionally, Yosida et al. (2019) inferred that compared to autotrophic angiosperms, DNA translocation occurs more frequently from host to parasite, indicating that this process may have played a significant role in the parasite’s evolution and adaptation.

**Table 4:** Summary of horizontal gene transfer involving parasitic plants.

Plants	Target genes	Transgenes	Size	Functions	References
*Viscum scurruloideum*	*cytochrome c maturation* (*ccmB*)	mitochondrial gene	~700 bp	Cytochrome c maturation pathway membrane protein	(Skippington et al., 2015)
*Cuscuta gronovii*	*mttB*	mitochondrial gene	~780 bp	Mitochondrial inner membrane transporter	(Park et al., 2015)
*Orobanche aegyptiaca*	*strictosidine synthase-like* (*SSL*) gene	nuclear genome	~691 bp	Might perform functions in haustorial formation and vegetable growth.	(Zhang et al., 2014)
*Phelipanche aegyptiaca*	*albumin 1* gene	nuclear genome	∼800 bp	Providing a large pool of sulfur storage and exhibiting toxicity to insect herbivores in certain legumes	(Zhang et al., 2013)
*Panicum hallii*	*Panicum Chr03*	nuclear genome	100 kb	–	(Yoshida et al., 2019)

## 6 RNAs translocation in host-parasite

### 6.1 siRNA translocation

siRNA (small interfering RNA), ranging from 21 to 24 nucleotides in length, can spread through the phloem in plants and mediate gene silencing extensively within the plant system (Denli and Hannon, 2003). Studies have shown that siRNA not only propagates systemically within the plant but can also transfer between host and parasitic plants via phloem connections. Recent research indicates that siRNA has great potential in regulating the gene expression of parasitic plants and controlling the growth of parasitic weeds, particularly in the context of gene silencing mechanisms between host and parasitic plants. The specific details are shown in the **Table 5** below.

**Table 5:** siRNA-mediated gene silencing in parasitic plants.

Hosts	Parasitic plants	Target gene	Result	References
Transgenic lettuce (*Lactuca sativa*)	*Triphysaria versicolor*	A hairpin RNA containing a fragment of the GUS gene (hpGUS)	Silencing of the GUS gene in parasitic plants	(Tomilov et al., 2008)
*Medicago truncatula*	*T. versicolor*	The cytosolic acetyl-CoA carboxylase (ACCase) gene in hairpin conformation (hairpin ACCase)	ACCase transcript levels were dramatically decreased in T. versicolor	(Bandaranayake and Yoder, 2013)
Transgenic tomato or tobacco rattle virus (TRV)-mediated Nicotiana benthamiana	*P. aegyptiaca*	Endogenous PaACS (1-aminocyclopropane-1-carboxylate synthase), PaM6PR (Mannose 6-phosphate reductase), and PaPrx1 (Peroxidase)	PaACS, PaM6PR, PaPrx1 were reduced in parasite	(Dubey et al., 2017)

### 6.2 mRNA translocation

mRNA (messenger RNA) translocation is a common process between host and parasitic plants, occurring through parenchyma cells and phloem across haustorium junction (Westwood et al., 2010). This process involves bidirectional molecular communication, signal regulation and adaptive mechanisms (Park et al., 2021). The efficiency of mRNA transfer is dynamically regulated by several factors, including the host’s physiological status, the duration of haustorial connection, and the affinity between the host and the parasitic plant.

First, the host’s physiological condition plays a key role in determining the transport efficiency. A healthy host, with active metabolic signaling and a stable supply of resources, supports higher mRNA transfer rates. When the host is infected by pathogens, defense mechanisms such as the activation of the salicylic acid (SA) signaling pathway can inhibit the formation and function of the parasitic haustoria (Majumdar et al., 2023). Similarly, environmental stresses like water scarcity or nutrient deficiency also decrease the efficiency of haustorial connection, limiting mRNA transfer (Zhang et al., 2024). For example, in the root parasitism model between *Haloxylon ammodendron* (Chenopodiaceae) and *Cistanche deserticola* (Orobanchaceae), hydroponic experiments on the host revealed that a well-watered host supports a higher mRNA transfer (Fan et al., 2022).

The duration of haustorial connection also affects transfer efficiency. Longer connections allow for cumulative transfer and stabilization of molecular channels, ultimately improving transport rates. In the parasitic system between *Phtheirospermum japonicum* and *Arabidopsis*, the formation of vascular bridges by the haustorium takes several days. Initially, the connection is unstable, leading to low mRNA transfer rates (Wakatake et al., 2020). However, as the connection strengthens, particularly with the formation of xylem bridges, the transfer of mRNA gradually increases (Cui et al., 2025).

Another critical factor is the molecular affinity between the host and the parasitic plant. Higher molecular compatibility enhances the success rate of transfer by reducing conflicts and improving efficiency. When the parasitic plant is closely related to the host species—such as within the same family—molecular recognition mechanisms, like effector proteins, promote efficient RNA exchange. For example, the haustorium of *Triphysaria versicolor* (a hemiparasite) can silence host genes, and the efficiency of this process is largely dependent on the genomic similarity between the two species. If the genetic divergence is too large, cross-silencing fails (Yoshida et al., 2016).

Studies have shown that mRNA from hosts plants, such as tomato and *Arabidopsis*, can travel long distances from the haustorium and translocate into the parasitic plant *Cuscuta pentagona* (David-Schwartz et al., 2008; LeBlanc et al., 2013). Moreover, the mRNA translocation was documented in high numbers and in a bidirectional manner, both from host to parasite and parasite. Kim et al. (2014) reported that 45% (9518) of the total genes expressed in the host *Arabidopsis* transcriptome were detected in the parasite *Cuscuta*, while 24% (5973) of the genes expressed in *Cuscuta* showed strong evidence of translocation into *Arabidopsis*. In contrast, only 1.6% (347) of genes expressed in host tomato were detected in the parasite *Cuscuta*, and 0.8% (288 genes) of the genes expressed in *Cuscuta* showed strong evidence of translocation into tomato, both of which are fewer than those observed in Arabidopsis. These differences in mRNA translocation rates between *Arabidopsis*-*Cuscuta* and tomato-*Cuscuta* suggest that the translocation selectivity mechanisms might be host-specific.

### 6.3 miRNA translocation

In addition to siRNA and mRNA, microRNA (miRNA) is also highly mobile between host and parasite (Johnson and Axtell, 2019). Recent studies have shown that the haustorium of *Cuscuta campestris* can accumulate high levels of novel miRNAs while parasitizing *Arabidopsis thaliana*. Some of these miRNAs targeted *A. thaliana* mRNA, leading to mRNA cleavage, the production of secondary siRNAs, and a reduction in host mRNA accumulation. Additionally, the same miRNA was expressed and activated when *C. campestris* infected host *Nicotiana benthamiana*. These data shown that miRNA translocated from parasite act as trans-species regulators of host-gene expression, implying that they may function as virulence factors to facilitate the establishment of parasitism (Shahid et al., 2018).

## 7 Protein translocation in host-parasite

Several lines of evidence suggest that proteins can be translocated between host and parasite (Zhang et al., 2025). For example, Haupt et al. (2001) found that green fluorescent protein (GFP) could be translocated from the transgenic tobacco plants to the parasite *Cuscuta reflexa*. Subsequently, Birschwilks et al. (2007) demonstrated that *Cuscuta*, parasiting two type transgenic *Arabidopsis*, one encoding GFP (27 kDa) and the other encoding a GFP–ubiquitin fusion (36 kDa), could acquire GFP but not GFP–ubiquitin fusion. This finding implies that the size of translocated proteins between host and parasite may be specific. Furthermore, Jiang et al. (2013) found that phosphinothricin acetyltransferase (PAT) could translocate from the host soybean to the parasite *Cuscuta pentagona*, resulting in the herbicide resistance of *C. pentagona*. Interestingly, Shen et al. (2020) demonstrated that FT (Flowering Locus T) proteins synthesized in host plants (soybean or tobacco) could move into dodder stems, where they physically interact with a dodder FD transcription factor to activate dodder flowering.

Recently, Liu et al. (2020) found that hundreds to more than 1500 proteins were translocated between the parasite *Cuscuta* and the host *Arabidopsis* or soybean. Notably, hundreds of inter-plant mobile proteins were detected in the seeds of both *Cuscuta* and the host soybean. These proteins also retained their activity after long-distance translocation between plants. The types of proteins that have undergone migration are as shown in the **Table 6**.

**Table 6:** The protein types involved in the migration between the host and the parasitic plant.

Protein name	Function	Mol. weight [kDa]	References
Glyceraldehyde-3-phosphate dehydrogenase	Participate in the glycolysis pathway	∼43	(Liu et al., 2020)
AvrE1 (DspA/E)	Regulate host immune responses and physiology.	∼200	(Yuan et al., 2021; Nomura et al., 2023)
tobacco mosaic virus	A movement protein, promoting the transport of viral nucleic acids through plasmodesmata	∼30	(Kellmann, 2001)
Green fluorescent protein (GFP)	Function tracing	∼27	(Aly et al., 2011)

## 8 Phytohormone translocation in host-parasite

Phytohormone, which plays key roles in numerous physiological and developmental process in plant, can translocate between host and parasite. The transfer of plant hormones between parasitic plants and their hosts is not limited to a single hormone, but rather involves a complex network of multiple hormones working together for coordinated regulation. Plants regulate hormone response pathways at multiple levels, including biosynthesis, metabolism, perception, and signaling (Anfang and Shani, 2021). **Table 7** shows different phytohormone types between host and the parasitic plant.

**Table 7:** The phytohormone types involved in the migration between the host and the parasitic plant.

Phytohormone type	Function	Interaction	Reference
Strigolactones (SLs)	A class of carotenoid-derived phytohormone with diverse functions in plant growth and development	Initially discovered as host root-derived germination stimulants in rootparasite *Striga* spp. and *Orobanche* spp.	(Cook et al., 1966)
Cytokinin	Induce changes in gene expression related to cell division and differentiation, ultimately modifying host root morphology and affecting host fitness.	Translocat between both the host *A. thaliana* and hemiparasitic plant *Phtheirospermum japonicum*.	(Spallek et al., 2017)
Auxin	Parasitic plants secrete auxins which affect the division of host cells, inducing the formation of structures (such as nutrient cells) that are conducive to parasitism.	Plant-parasitic nematodes promote the development of feeding sites by manipulating the host’s auxin homeostasis.	(Oosterbeek et al., 2021)

## 9 Other molecules translocation in host-parasite

Using the parasite C*uscuta* as a bridge connecting different host plants within a “microcommunity”, Hettenhausen et al. (2017) found that when one host plants was treated with insect feeding, the systemic insect resistance signal generated by the feeding leaves was transferred to other host plants in the “microcommunity” through *cuscuta*. This transfer induced transcriptome and metabolite responses of other host plants, thereby enhancing their insect resistance.

*Cuscuta* can transmit insect feeding-induced signals between connected plants. When plants are subjected to insect herbivory, they activate both local and systemic defense responses, such as the jasmonic acid (JA) signaling pathway, and produce defense metabolites, including proteinase inhibitors (TPIs). These signals are rapidly transmitted and can spread over long distances (Hettenhausen et al., 2017). Zhou et al. (2023)investigated the differentially expressed genes (DEGs) in white clover under *Cuscuta* parasitism and the defense pathways they are involved in. The study found that *Cuscuta* parasitism triggers a complex molecular defense response in white clover, including immune system reprogramming (R genes, PR proteins) and the activation of hormone signaling pathways (JA, SA, ABA), among others.

Using transgenic tomato and tobacco plants expressing calcium indicator proteins, Albert et al. (2010) detected calcium ion influx at the site of haustorial penetration early during *Cuscuta* contact with the host. They concluded that calcium signaling is one of the early plant responses in recognizing the parasitic invader. After detaching from its host, *Cuscuta* spp. coordinates the balance between basal degradation and apical growth through the regulation of calcium signaling and cell structural proteins (Zhang et al., 2021)

## 10 Conclusions

The interaction between parasitic plants and their host plants holds significant ecological and evolutionary importance. First, parasitic plants acquire water, nutrients, and organic molecules from their hosts through specialized structures such as haustoria, regulating material cycles and driving the co-evolution of symbiosis and competition between plants. Second, the genetic integration and co-evolution between parasites and hosts reflect the long-term adaptation processes between species, contributing significantly to the competition and adaptive evolution within ecosystems (Estioko et al., 2014; Fei et al., 2021). Parasitic plants exhibit marked path dependence on their hosts, and through specific signaling mechanisms, they promote the co-evolution of symbiosis and competition among plants (Oñate and Munné-Bosch, 2009). In-depth research into the mechanisms of parasitic plant-host interactions will enhance our understanding of plant adaptive evolution and provide theoretical support for agricultural control strategies, crop protection, and plant breeding.

Unraveling the routes and functional significance of translocated molecules remains a central challenge in plant-parasite research. The translocated routes of small molecules, such as nutrients and herbicides, are phloem or xylem potentially, but the pathways governing macromolecular trafficking (DNA, RNA, proteins) are only partially resolved. DNA translocation in host-parasite is considered a historical event, typically occurring over extended evolutionary periods between the parasite and its host, which is different from DNA translocation in plant grafting system. Current research suggests that the scale of DNA translocation is limited and may occur in isolation. However, whether DNA translocation in host-parasite interactions is a random or selective event remains unclear and warrants further investigation. Additionally, the functional significance of this DNA translocation requires further exploration.

**Figure 1:**
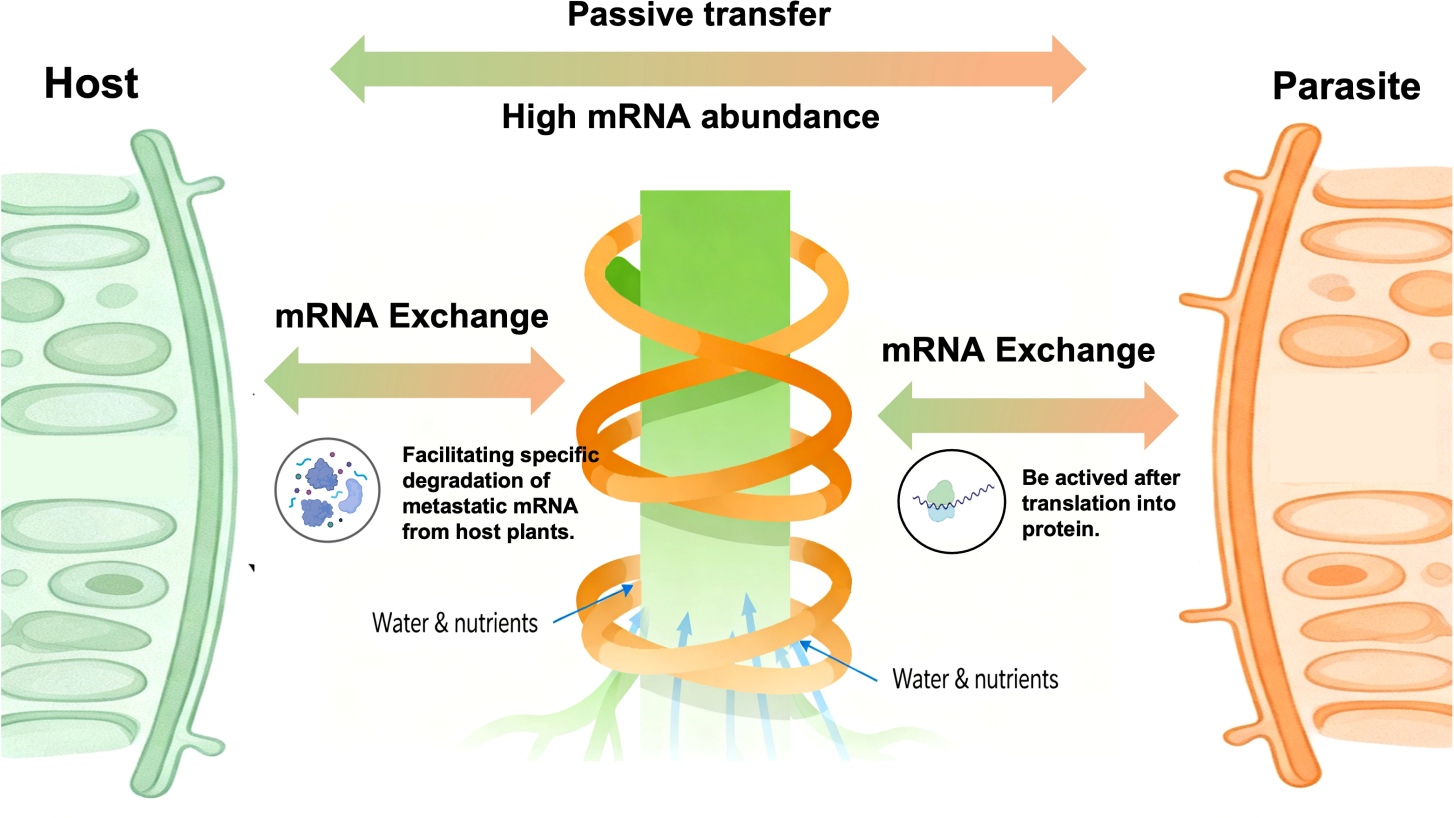
The fate of translocated mRNA.

In contrast to the DNA translocation, RNA translocation in host-parasite interactions occurs on a much larger scale. The translocated RNA mainly refers to mRNA, which carries sequence-specific information, as others may be translated into proteins that precisely regulate gene expression, or they may degradation through cellular autonomous or involuntary processes. Consequently, a key concern is a understanding the ultimate fate of the translocated mRNA once it reaches its destination. Three hypotheses have been proposed regarding the fate of translocated mRNA (**Figure 1**): (i) One hypothesis suggests that translocated mRNA provides nutrients to the parasite by facilitating specific degradation of metastatic mRNA from host plants, utilizing mechanisms that accurately distinguish exogenous mRNA from parasite’s own mRNA. (ii) One posits that the most important role of translocated mRNA is to be active after translation into protein, thereby influencing the metabolic pathways of parasitic plants or participating in the regulation of gene expression. (iii) The other hypothesis suggests that the abundance of mRNA appears to be the factor. The higher the mRNA abundance, the more likely it is to undergo passive transfer. Additionally, the haustorium serve as the key pathway for mRNA exchange (Westwood and Kim, 2017; Fan et al., 2022).

Future research will integrate physiological, molecular biology, and omics technologies to systematically explore the mechanisms of material exchange, gene regulation, and signal transduction between parasitic plants and their host plants. Physiological methods, such as ion and water exchange measurements and metabolite analysis, will reveal how parasitic plants utilize host resources, while molecular biology techniques, such as RNA sequencing and gene editing (CRISPR-Cas9), will help deepen our understanding of the role of key genes and their involvement in the parasitic process. Omics technologies, including genomics, metabolomics, and proteomics, will provide a comprehensive perspective on the interactions between parasitic plants and their hosts, particularly in the areas of metabolic regulation and signaling pathways. Furthermore, research on mutants and transgenic plants will offer new insights into the genetic basis of parasitism. The integrated application of these advanced technologies will provide a novel theoretical framework for uncovering the molecular mechanisms underlying parasitic plant-host interactions, offering significant practical value for agricultural and ecological management.
